# Self-perceived views on offender rehabilitation in detained adolescent boys: a qualitative analysis in the context of the good lives model

**DOI:** 10.3389/fpsyg.2023.1153093

**Published:** 2023-05-19

**Authors:** Colinda M. B. Serie, Corine De Ruiter, Stefaan Pleysier, Johan Put

**Affiliations:** ^1^Leuven Institute of Criminology, Faculty of Law, KU Leuven, Leuven, Belgium; ^2^Faculty of Psychology and Neuroscience, Maastricht University, Maastricht, Netherlands

**Keywords:** GLM, offender rehabilitation, adolescents, treatment motivation, well-being

## Abstract

**Introduction:**

An upcoming offender rehabilitation model, the Good Lives Model (GLM), proposes that effective offender rehabilitation should adopt a *dual* focus: reducing recidivism risk *as well as* enhancing the offender’s well-being. To achieve this, the GLM suggests rehabilitation should include the prosocial fulfilment of a universal set of human needs termed “primary goods.” A focus on primary goods attainment and well-being is hypothesized to improve treatment motivation and achieve more sustainable desistance from future offending. Although this model sounds promising, empirical evidence for these assumptions is limited, especially among youth.

**Methods:**

Twenty Flemish and Dutch detained adolescent boys (14 to 17 years old at the time of their arrest) were interviewed during their detention using a semi-structured interview. They were asked about their well-being, needs and goals during rehabilitation, their treatment motivation, and their views on recidivism and rehabilitation.

**Results:**

The results show that a match between the boys’ well-being needs, and the treatment goals set in collaboration with the institution could improve treatment motivation and rehabilitation efforts. The boys also mentioned other factors with a positive impact on their treatment motivation: increased levels of freedom and autonomy; having a future (prosocial) perspective; investing in a therapeutic alliance; and, working on individual factors (i.e., improving coping skills, school or work skills, and relationships with prosocial friends and family).

**Discussion:**

These factors closely align with working on the GLM primary goods of “excellence in work and play,” “excellence in agency,” and “relatedness,” which can be helpful in enhancing well-being and treatment motivation in offender rehabilitation.

## Introduction

A common challenge in (young) offender rehabilitation is its limited effectiveness, expressed as high treatment dropout and high recidivism rates, partly as a result of low treatment motivation (Ginsburg et al., 2002; Howells and Day, 2003, 2007; McMurran and Ward, 2004, 2010; Drieschner and Verschuur, 2010; Olver et al., 2011; Carl et al., 2020). Detained adolescent offenders in particular, may experience low levels of motivation concerning the mandatory interventions imposed upon them by legal authorities (Harder et al., 2015; Fortune, 2018; Carl et al., 2020).

Interventions imposed by (juvenile) justice systems often rely on the Risk Need Responsivity (RNR) model of offender rehabilitation (Andrews and Bonta, 2010). This model states that in order to effectively reduce recidivism, interventions should primarily focus on criminogenic risk factors that are directly linked to delinquency and recidivism (e.g., antisocial personality and attitudes, criminal peers, family/family problems, school/work problems, lack of constructive leisure activities, and substance abuse) (Andrews and Bonta, 2010). In the beginning of the century, Tony Ward and colleagues criticized the RNR model for its sole focus on diminishing risk factors and developed the “Good Lives Model of offender rehabilitation” (GLM; e.g., Ward and Stewart, 2003a,c; Ward and Maruna, 2007; Yates et al., 2010; Ward and Fortune, 2013; Purvis et al., 2015; Barnao et al., 2016).

The GLM states that effective interventions should not only focus on risk factors, but also on strengthening the offender’s skills to meet personally relevant human needs that improve well-being and quality of life (Ward, 2002a,b; Ward and Fortune, 2013). Working towards a better life could reduce the risk of recidivism more sustainably by promising a happier and prosocial life, rather than merely a less risky one. Furthermore, the model assumes that focusing not only on risk factors determined by professionals, but also on personally relevant needs (so-called “Primary Goods”) and well-being of the offender, will contribute to greater treatment motivation and subsequently reduce the risk of recidivism (Ward and Stewart, 2003a,c; Ward and Fortune, 2013). This makes the GLM, which has its roots in positive psychology, a capabilities- or strengths-based approach (Ward and Mann, 2004; Ward and Gannon, 2006; Ward and Maruna, 2007). It combines improvement of well-being (strength) with reduction of recidivism (risk), resulting in a hybrid and holistic rehabilitation model (Ward, 2002b; Ward and Maruna, 2007).

The GLM is based on two - largely theoretical - etiological assumptions. The *first etiological assumption* states that all human beings strive to obtain, to varying extents, a universal set of human needs and goals to achieve a higher sense of well-being (Ward, 2002a,b; Ward and Stewart, 2003a; Ward and Maruna, 2007). In the GLM these needs and goals are termed 11 “*primary goods*”: (healthy and safe) “life”; “knowledge”; “excellence in work”; “excellence in play”; “excellence in agency”; “inner peace”; “relatedness” with friends, family, or a partner; (being part of a) “community”; “spirituality” (seeking purpose and meaning in life); “creativity”; and “pleasure” (Ward, 2002a,b; Purvis et al., 2015). The *second etiological assumption* of the GLM is that a failure to prosocially obtain primary goods needed for well-being, can result in criminal behavior (Ward and Stewart, 2003a; Ward and Maruna, 2007). All human actions, including criminal or antisocial acts, are viewed as ultimately linked to the pursuit of human goods. Criminal behavior, according to the GLM, can thus be seen as an alternative, antisocial attempt to obtain primary goods when an individual fails to secure their primary goods in a prosocial way (Ward and Stewart, 2003a; Ward and Gannon, 2006; Purvis et al., 2011, 2015).

In this line of reasoning, the GLM views the RNR’s criminogenic needs or risk factors as the internal (i.e., *personal limitations*) or external (i.e., *environmental disadvantages*) obstacles that interfere with an individual’s capacity to achieve their primary human goods in a prosocial way (Ward, 2002b; Ward and Stewart, 2003a,b,c; Ward et al., 2006; Purvis et al., 2011).

Based on these etiological assumptions, the GLM argues that effective rehabilitation interventions should thus combine the reduction of recidivism (risk) with an improvement in the offender’s personal well-being (Ward, 2002b; Ward and Stewart, 2003a; Ward et al., 2006; Ward and Gannon, 2006; Ward and Maruna, 2007; Ward and Fortune, 2013). Understanding the offenders’ behavior as a function of achieving one or more primary goods, can guide interventions to help them secure goods that are important to them in a socially responsible manner. Moreover, working toward personally relevant goals and well-being, instead of merely working to reduce risk factors determined by professionals, is believed to increase treatment motivation and further reduce recidivism risk (Ward, 2002b; Ward and Stewart, 2003a; McMurran and Ward, 2004; Ward et al., 2006; Ward and Gannon, 2006; Ward and Maruna, 2007; Laws and Ward, 2011; Ward and Fortune, 2013). In comparison to risk-only perspectives, Polaschek (2012) argues that:

*One of the most useful aspects of critiques from strength emphasizing perspectives is in reminding us of the importance of giving offenders reasons to want to engage in desistance and change* (e.g., Porporino, 2010; Ward and Maruna, 2007), *not just the capacities to do so* (p. 8).

Initially, the GLM was developed for adult offenders, but it is assumed that it can also be used as a rehabilitation model for adolescents (Fortune, 2018). Over the years, an increasing number of GLM-based interventions are being developed for juvenile offenders (e.g., Print, 2013; Wainwright and Nee, 2014). However, it could be problematic to apply the GLM assumptions and principles, developed for adults, to young people without substantiation. That is, although the GLM seems promising in theory, little empirical research exists examining the underlying etiological assumptions, the applicability, and the effects of this model (but see e.g.,; Bouman et al., 2009; Netto et al., 2014; Van Damme et al., 2016; Barendregt et al., 2018; Loney and Harkins, 2018; Ryan et al., 2019; Mallion et al., 2020; Serie et al., 2020, 2021; Zeccola et al., 2021; Serie, 2022).

Research that assessed the GLM’s primary goods attainment in adult offender rehabilitation revealed that desisting adult (sex) offenders strive for all primary goods to some extent (Harris et al., 2019, based on 42 interviews; Lindsay et al., 2007; based on two case studies; Whitehead et al., 2007; based on one case study; Willis and Ward, 2011, and based on 16 interviews). Barnett et al. (2014) analyzed the differences in treatment change between a risk-focus and a primary goods-focus in offender rehabilitation for adult sex offenders. One group was treated with a traditional relapse prevention program (*n* = 321) targeting risk factors only, while the other program (*n* = 202) was based on the GLM. The latter focused not only on risk factors, but also on building motivation for a better life, including identification of the primary goods that group members wished to acquire. Although they found few differences between the two programs, those who completed the GLM-based program showed more improvements regarding their child abuse attitudes. Furthermore, a larger proportion of those who completed the GLM-based program attained a “treated profile,” which meant their psychometric assessment scores (on personal distress, coping skills, self-esteem, empathy, loneliness, assertiveness, locus of control, and child abuse attitudes) were indistinguishable from those of a group of nonoffenders. This finding, however, did not take into account that those who completed the risk-focused program showed more dysfunctional scores pre-treatment (Barnett et al., 2014).

Similarly, Harkins et al. (2012) assessed two rehabilitation programs for sex offenders: one relapse prevention intervention focused on risk reduction (*n* = 701) and one GLM-based intervention (*n* = 76). Despite the large difference in sample size between the two groups, the authors concluded they found no differences in the attrition rates or the rates of treatment change between the two programs, indicating they were equally effective. Nonetheless, participants of the GLM-based intervention (based on 15 interviews) reported to be more optimistic and opportunity-focused compared to the participants of the risk-focused relapse prevention intervention (based on 5 interviews; Harkins et al., 2012).

More recently, Ryan et al. (2019) examined eight adult male sex offenders who were still participating in a community GLM-based treatment and five men who had completed the treatment. The participants mentioned the therapeutic alliance, improved treatment engagement as they realized addressing their difficulties resulted in a happier offense-free life, addressing non-criminogenic needs, and understanding the root causes of their offending behavior as the essential elements in their process of change during treatment. Despite these promising preliminary results, they are predominantly based on qualitative studies in (very) small samples of male adult (sex) offenders.

For detained young offenders, empirical research is even more scarce (Netto et al., 2014; Fortune, 2018; Mallion et al., 2020; Zeccola et al., 2021). Two studies evaluated the assumptions of the GLM with, respectively, delinquent girls (Van Damme et al., 2016) and boys (Barendregt et al., 2018) in a residential institution. However, the results from these studies contradict each other. Barendregt et al. (2018) found no relationship between quality of life (as a proxy measure for well-being, measured as a combination of satisfaction with social participation, health, family relationships, living situation, safety, finances, self-esteem, and purpose in life) at admission and delinquent behavior 12 months after discharge. In contrast, Van Damme et al. (2016) found that a lower quality of life at admission (measured as a combination of satisfaction with physical health, psychological health, social relationships, and living environment), albeit indirectly through mental health problems, was found to be related to higher recidivism 6 months after discharge. These studies did not assess the role of treatment motivation.

Recently, Serie (2022) examined within-person changes in self-reported psychopathological problems, primary goods satisfaction, subjective well-being, and treatment motivation during detention (from the start of detention to three and a half months later). Based on longitudinal data from 63 Flemish and Dutch detained adolescent boys (ages 14 to 18), we found that improvement in primary goods satisfaction increased adolescents’ well-being over time, which in turn enhanced their treatment motivation and (subsequently) lowered their recidivism risk. However, the question whether all primary goods and/or other factors can increase adolescents’ well-being and treatment motivation during detention, remains unanswered.

Especially the period of adolescence (between the ages of 12 and 18 years) has long been known as a turbulent phase of life, with a greater risk of mental and behavioral problems due to the many mental, physical, and social changes (Arnett, 1999; Žukauskiene, 2014). During this period of development and change, adolescents are exploring their own identity and are starting to build their own life and future (Zirkel, 1992; Žukauskiene, 2014; Ryan and Deci, 2017b). Accordingly, studies have found that adolescents pursue goals that are unique to this particular developmental phase (Nurmi, 1991; Massey et al., 2008; Messersmith and Schulenberg, 2010). The type of goals that people strive for can vary greatly between individuals, influenced by personal, cultural, and social contexts. In general, however, adolescent goals seem to follow a “cultural prototype” in which they are expected to achieve educational goals first, followed by occupational and relationship/family goals. Additionally, social needs become more important when adolescents gain independence from their parents and spend a greater amount of time in the company of peers (Nurmi, 1991; Massey et al., 2008; Messersmith and Schulenberg, 2010). Hence, it is quite conceivable that adolescents prioritize other GLM primary goods than adults.

Moreover, studies to date have not yet explored the self-perceived views of detained youth on the hypothesized associations between all primary goods, well-being, treatment motivation, and recidivism risk.

Although quantitative questionnaires and measures exist that assess offenders’ needs and goals that can be related to treatment motivation (see, e.g., Campbell et al., 2010; Harper et al., 2020), they use structured and predefined variables. Furthermore, while quantitative research provides an overall picture of associations at the group level, it cannot be applied automatically to the individual case. Moreover, well-being and the primary goods refer to broad theoretical constructs difficult to capture in a limited number of survey items. On the other hand, qualitative studies allow us to discover the youths’ personal views of their experiences during detention (Silverman, 2013; Mortelmans, 2020).

Therefore, qualitative research could expand our knowledge on the ideas, insights, and narrative explanations of detained youth themselves (Horstkötter et al., 2012). Specifically, qualitative data can provide insight into how detained adolescents view their own needs, well-being, treatment motivation, and rehabilitation process during their stay in a residential institution. Hence, in the current study, we conduct a qualitative analysis of the GLM’s assumptions about primary goods satisfaction and well-being and its effects upon treatment motivation and young offender rehabilitation, using interviews conducted in a sample (*N* = 20) of detained adolescent boys.

## Method

### Setting, sampling, and study procedure

The current study^1^ was part of larger mixed-method study examining the assumptions of the Good Lives Model in adolescents (Serie, 2022). The study was conducted in three all-boys secure youth detention centers: two in Flanders, Belgium and one in the south of the Netherlands (Limburg). The Flemish and Dutch centers were similarly characterized by restrictive infrastructures (i.e., aggravated locked doors, high fences, and barred windows) and daily regimes (i.e., strict rules, scheduled activities, limited and scheduled contact with close relatives). The centers focus on rehabilitation and reintegration treatment (including aggression regulation therapy and addiction treatment) where juveniles are gradually granted more liberties and periods of leave.

The sample of detained adolescents from the larger study (*N* = 170) was asked by the first author (an outsider of the institutions) if they wanted to participate in a qualitative interview for the current study. Eighty-two (48.2%) of the sample refused participation in the interview and 88 (51.8%) agreed to participate at T1 and signed the informed consent. Those who rejected usually did so because of time restrictions or they lacked concentration to continue. Of these 88 consenting participants, almost half (*n* = 42; 47.7%) were randomly selected to participate in the additional interviews. The participants were interviewed at three points in time during their detention: first (T1), approximately 2 weeks after their arrest; next, at T2, approximately 3 months later, and finally at T3, which was again approximately 3 months later. Because of time restrictions and early discharges, not all participants were interviewed at each of the three time points.

The current study focuses on the interviews conducted at T2 and T3. We chose both assessment points as we aimed to study whether and how the boys’ views shifted over time (where possible) between T2 and T3. At T1, the detained adolescents were interviewed about their primary goods attainment and its relationship to their well-being and offending behavior before their arrest. At T2 and T3, participants were interviewed using a semi-structured interview (see Appendix A) focusing on the experiences of the adolescents during their detention. As this study is focused on the assumptions of the GLM about the role of primary goods and well-being and motivation during detention, T1 data about the period before arrest was excluded from the current analyses. As a result, at T2 and T3 the youth were asked about their well-being, needs and goals during rehabilitation, their treatment motivation, and their views on future recidivism and rehabilitation. The interviews ranged in duration from 10 to 45 min with a mean of 20 min. The interviews were transcribed verbatim by a researcher who pledged confidentiality in accordance with ethical and privacy guidelines. Although the interviews were originally held and transcribed in Dutch, the quotes included in the Results section of this paper were translated into English (the original Dutch quotes are added in the accompanying footnotes).

Ultimately, 20 of the 42 detained adolescent boys who participated at T1, also participated at T2, at T3, or both. At T2, 16 boys were interviewed, 8 of whom only participated at T2 and another 8 also participated at T3. At T3, 12 youth were interviewed, 8 who had also participated at T2 and 4 who only participated at T3. Interviews with these 20 boys resulted in 28 interview transcripts. For those youth who participated at both T2 and T3 we aimed to compare their results over time.

### Qualitative analysis

The data of the 28 verbatim interview transcripts were analyzed using thematic content analysis (Braun and Clarke, 2006; Mortelmans, 2020). Thematic analysis is a qualitative approach for analyzing and reporting patterns or themes within text data. This type of analysis can take the form of an inductive as well as a deductive approach. An inductive approach means that the themes are derived from the data in a bottom-up fashion. This type of analysis is exploratory in nature and ensures that the results closely reflect the statements, experiences, and perceptions of the participants. Although we aimed to explore the youth’s ideas and experiences, with little suggestion from the interviewer, the current study also focuses on examining the GLM. As a result, the data are not analyzed in a theoretical and epistemological vacuum, but will be analyzed in the context of the GLM.

Qualitative research software (NVivo 1.4.1, QSR International, 2021) was used to organize the verbatim interview transcripts. The transcripts were analyzed line-by-line using the iterative bottom-up (i.e., inductive) approach as described by informed grounded theory and thematic analyses (Glaser and Strauss, 1967; Thornberg, 2012; Creswell and Poth, 2016; Mortelmans, 2020). First, concepts were identified in codes during an open coding phase. Examples of codes that emerged in this phase included: specific situations, experiences, ideas, and people that were mentioned as important to the youths’ well-being and motivation during detention, as well as specific self-perceived needs, obstacles, and problems they experienced during rehabilitation. After the first round of coding, all interviews were analyzed again to check whether there were additional quotes belonging to the codes that had emerged in the first round. This inductive approach allowed the identification of recurring categories and concepts voiced by the youth and ensured that their own words and expressions were preserved in the results (Braun and Clarke, 2006; Mortelmans, 2020). The categories that emerged consisted of: well-being needs, treatment goals, factors affecting treatment motivation and rehabilitation, antisocial ideas, and prosocial ideas.

In a next step, we analyzed the emerged codes in a deductive manner as described the “theoretical thematic analysis” (Braun and Clarke, 2006; Mortelmans, 2020). That is, the categories that had emerged in the previous inductive approach were examined, compared, and -when possible- categorized in terms of the theoretical higher-order themes of the GLM: the importance and pursuit of the eleven primary goods for well-being, treatment motivation, and rehabilitation. This resulted in a thematic framework in which the views of the detained adolescents could be tested against the theoretical assumptions of the GLM. More specifically, this technique allowed for the translation of the words and perspectives of the detained adolescents in the context of the GLM assumptions.

### Sample characteristics

At T2, the 20 adolescent boys ranged between 14 and 18 years old and had a mean age of 16.50 (*SD* = 0.95). A little more than half (*n* = 12, 60%) attended school before they were arrested, 25% (*n* = 5) were temporarily suspended from school, and 15% (*n* = 3) did not attend school and were also not employed. Of those who attended school or were only temporarily suspended (*n* = 17), 29.4% (*n* = 5) attended vocational secondary education, 35.3% (*n* = 6) attended part time secondary education (combined with part time employment and/or a traineeship), two (11.8%) attended special secondary education, two (11.8%) attended technical secondary education, one (5.9%) attended general secondary education, and one (5.9%) attended higher education. The majority of the participants were born in Belgium (*n* = 15, 75%), two (10%) were born in the Netherlands, one (5%) in Italy, one (5%) in the Netherlands Antilles, and one (5%) in Surinam. The majority of the participants’ biological parents were divorced (*n* = 16, 80%), while for 20% (*n* = 4) their parents lived together. Before their arrest, 20% (*n* = 4) lived together with both their parents, 40% (*n* = 8) lived with their mother most of the time, one (5%) lived with his father most of the time, one (5%) lived with both divorced parents, 15% (*n* = 3) lived in a residential institution, and 15% (*n* = 3) lived somewhere else (i.e., with foster parents, friends, or alone).

Most boys reported to have conducted several delinquent behaviors in the 12 months before their arrest. They reported to have conducted: vandalism (*n* = 15; 75%), weapon carrying (*n* = 12; 60%), shoplifting (*n* = 14; 70%), theft (*n* = 14; 70%), and physical assault (*n* = 16; 80%). None of the participants reported sexual offences. On average, the boys were detained in total for 7.5 months, ranging from 4 to 18 months.

## Results

### Well-being needs

We aimed to uncover the detained boys’ well-being needs by asking what was important in their lives, both while being detained and after their release. Their answers revealed several recurring needs during detention related to: relationships with their family, friends, and partner (i.e., the primary good of “relatedness”); pursuing goals related to work, school, and leisure (i.e., the primary goods of “excellence in work” and “play”); experiencing the freedom to pursue one’s own goals (i.e., the primary good of “excellence in agency”); mental health and substance use (i.e., the primary good of “inner peace”); and basic physical needs, such as having a place to live and having enough money to provide for oneself (i.e., the primary good of “life”). In what follows, we will discuss each of these needs in more detail.

#### Relatedness with family, friends, and partners

When the boys were asked what was important in their lives while being detained, almost all referred to the need to connect (again) with their loved ones.

“*Talking to my father. Just being at home…*”^2^ (Participant 10 at T3, 17 years old)

“*The weekend visits, or when my… my girlfriend comes to visit… Then I forget… forget all the rest*.”^3^ (Participant 2 at T3, 17 years old).

“*Hm… Just the bond with my family… I hope that will improve. And when I'm back home it will go well. The bond with my father I would also like to improve. Uh… It was his birthday last Sunday… I wanted to call him, but apparently, I now have a restraining order against him*.”^4^ (Participant 3 at T2, 16 years old)

It was not uncommon that the relationship between the youth and their family, especially parents, had been disrupted. In these cases, the boys usually explicitly mentioned the value of restoring this bond over time.

*“[I am happy…] now, when I'm at home or, uh, when I'm… seeing my friends again. Uh… Yeah. Now that's… Now it is strange for me to say that because before [being detained] I would never say ‘[I am happy…] when I see my mother happy', for example.”*^5^ (Participant 3 at T3, 16 years old)

#### Excellence in work and play

Next, to relationships, most boys expressed that doing well in school and finding a proper job (in the future) were important to them.

*“I just want to go to school… Because I know that I can and I know that I need that to prove myself to the court*.”^6^ (Participant 120 at T2, 17 years old)

*“I think the only thing that's going well is that I'm still enrolled in school. That I didn't get suspended.”*^7^ (Participant 148 at T2, 16 years old)

Also mentioned, but less often, was the need to participate in hobbies, such as going to the gym and playing soccer.

*“I’m thinking about playing at another soccer club or well, just playing soccer when I'm out of here*”^8^ (Participant 147 at T2, 17 years old)

#### Excellence in agency: freedom and future plans

While being detained, most boys expressed the value of being free. Especially in the beginning of their detention (before T2) they experience a complete lack of freedom. During their stay they gradually gain more liberties and periods of leave. Compared to T2, at T3 the boys experienced more freedom and more often were allowed to go on leave outside the institution. For some (especially those at T2 compared to T3), the need to be free was related to the need to have fewer restrictions and rules within the institution.

Participant: “*… there should just be an open facility.*” Interviewer: “*How would that help you think? The facility being more open?*” Participant: “*Because no kid or youngster wants to be locked up*.”^9^ (Participant 58 at T2, 14 years old)

“*Just less… less rules. More freedom.”*^10^ (Participant 148 at T2, 16 years old).

For others, especially those at T3 who were going towards to the end of detention, the need to be free was strongly related to the need of leaving the facility and return to their own lives.

“*I just want that uh… I'm free again, I just want to that day… I just want to enjoy that I'm free. And I don't want uh, how do you say that, that I don't want people to say to me 'at half past nine you have to be in your cell'*.”^11^ (Participant 25 at T3, 18 years old).

Again for others, freedom could be found in making one’s own choices for the future.

“… *more important is making your own choices. Defining your own life.”*^12^ (Participant 27 at T3, 16 years old).

However, as they are still minors, the boys’ level of agency and autonomy will always be more restricted compared to that of adults.

“*But in any case, there is still always someone who can make decisions about you, so… You have to listen to them and also be able to make decisions yourself*.”^13^ (Participant 5 at T2, 16 years old)

#### Inner peace: mental health and substance use

The majority of participants also expressed the importance of their mental health. In the period before their arrest, they experienced feelings of stress, anger, frustration, grief, and/or depression. Some tried to cope with these negative feelings by using substances.

“*When you smoke [weed]… then nothing will bother you so to speak. Well not nothing… but less*.”^14^ (Participant 2 at T2, 17 years old).

During detention, some boys expressed the need to stop using substances in order to live a happy life after their release.

Interviewer: “*You said you want a good life when you get out, what do you mean by that?*” Participant: “*That I will be able to stay off the drugs*.”^15^ (Participant 60 at T2, 17 years old).

#### Life: basic needs

As their release date drew closer, finding a place where the boys would be living next became increasingly important. While some would return to living with their parent(s), others would move to another residential institution or would be living on their own with support. Especially in the latter case, finding a proper job for a stable income to provide for one’s basic needs was very important to them.

“*I want to get my degree. And uh, now that I'm going to live on my own, I also uh, I also want to work and stuff*.”^16^ (Participant 164 at T2, 17 years old)

However, for some, money to provide for one’s basic needs was not enough and they aimed for a life of pleasure, riches, and luxury (i.e., the primary good of ‘pleasure’).

“*If you're rich, you can do more things. Pay for more things, just… Then you live a little more luxurious than… a normal person.*”^17^ (Participant 58 at T2, 14 years old).

### Treatment goals

Interestingly, the boys’ treatment goals, set by themselves and/or the institutional staff, usually corresponded to the needs they mentioned in the interviews. Their answers revealed several recurring treatment goals related to: relationships with their family, friends, and partner (i.e., the primary good of “relatedness”); pursuing goals related to work, school, and leisure (i.e., the primary goods of “excellence in work” and “play”); mental health and substance use (i.e., the primary good of “inner peace”); and having a place to live after their release (i.e., the primary good of “life”). Additionally, treatment goals referred to improving their behavior in general, taking responsibility for, and learning about the consequences of their choices (related to the primary good of “excellence in agency”). In what follows, these treatment goals will be described in more detail.

#### Relatedness with family, friends, and partners

During detention, the boys gradually gained more periods of leave where they could go home to their family, friends, and/or partner. Despite being detained and away from home for several months, most boys worked on their relationships with family as part of their treatment goals.

“*Yes, yes I have to now, well work on that… uh… I have to go home on day leaves and such.*”^18^ (Participant 2 at T2, 17 years old)

“*And uh, then on the weekends with those day-visits, for example, I don't, don't hang out with friends or anything. I uh, just try to be with my parents as much as possible. …well, I'm not always with my parents, I'm also often alone with my girlfriend, but then I try to do something with my, uh, with my mother or something. Like last weekend, I went out to dinner with my mother and my girlfriend*.”^19^ (Participant 60 at T2, 17 years old)

Regarding their friends, most boys wanted to and/or were stimulated to avoid their (antisocial) friends who have a bad influence on them. Avoiding certain friends, however, was considered a difficult task for the boys, especially when they had known them for several years.

“*I would like to push them aside and start over with new friends, but… that’s not so easy because I have known them… many friends, for four, five years*…”^20^ (Participant 133 at T2, 17 years).

#### Excellence in work and play

About half of the boys did not attend school (anymore) before their arrest. Especially for them, but also for most boys, getting back to school was an important treatment goal. Usually, they had set these goals for themselves to at least finish secondary school. After secondary school, some had the goal to attend higher (vocational) education, while others wanted to start working.

“*Uh, important [for me]… well yes, that I finish my school. Finishing school and finding a job.*”^21^ (Participant 25 at T3, 18 years old).

The need for leisure activities was mentioned less often, and in line with this, finding or participating in leisure activities was also mentioned only twice as a specific treatment goal.

#### Excellence in agency: behavior, responsibility, and consequences

Next, to relationships and school/work, the most often mentioned treatment goal set by the institution and mentioned by the boys themselves was improving their behavior. The boys learnt about the consequences of their (past) behavior and to take responsibility for their choices and actions. More specifically, their goals included being less impulsive and aggressive. They were taught alternative behavioral responses in challenging situations.

“*[I am working on]… uh, how to best respond to bad situations. Instead of attacking, how to find a better solution without attacking*.”^22^ (Participant 19 at T2, 17 years old).

“*That I… learn to think about the things that I could have done better and differently in the past… and that I need to do them differently in the future. And, that you have to think about that here too, because yes, you're here [in detention] for that*.”^23^ (Participant 120 at T2, 17 years old)

#### Inner peace: mental health and substance use

Many of the youth in detention mentioned they suffered from mental health problems, lack constructive coping mechanisms, and used substances as a coping mechanism instead.

“*When I feel bad it shows in my behavior, but I don’t really talk about it.*”^24^ (Participant 133 at T2, 17 years old).

“*When you smoke [weed]… then nothing will bother you so to speak. Well not nothing… but less*.”(see text footnote 14) (Participant 2 at T2, 17 years old).

During their detention the boys were taught alternative coping skills, such as talking with others and mental health professionals about their problems. This was offered within the institution and/or the boys were referred to therapy elsewhere.

“*…just by talking to the staff, social services, and the psychologist and such, talking about ‘look this is what I have done… [talking about] what is going wrong in my life…*’”^25^ (Participant 60 at T2, 17 years old)

#### Life after release

When the end of the detention period is near, the institution and the boys focused on where they would live after their release.

“*They [the institution] suggested that [assisted independent living], but I myself think that's important as well. When I'm eighteen or older that I can be independent*.”^26^ (Participant 133 at T2, 17 years old).

#### Factors affecting treatment motivation and rehabilitation

Four types of factors emerged from the interviews that – according to the boys themselves – could either diminish or improve their treatment motivation and affected their rehabilitation. The factors consisted of four types: (1) treatment goal factors, (2) autonomy and freedom, (3), staff factors, and (4) individual factors. The first type is related to how the boys perceive the ways in which they work on their treatment goals, particularly related to the needs for “excellence in work and play.” The second type included factors about the restriction or granting of freedom and autonomy within and outside the institution (i.e., the primary good of “excellence in agency”). Staff factors were related to the therapeutic alliance, communication, and interaction the boys have with staff members on the ward (i.e., related to the primary good of “relatedness”). Finally, individual factors consisted of factors related to boys’ emotional coping skills (i.e., the primary good of “inner peace”) and “relatedness” with their friends and family. We will now elaborate on these factors and on their role in the boys’ treatment motivation and rehabilitation.

#### Treatment goal factors: working on excellence in work, play, and pleasure

When asked how they felt about their detention period, different reactions emerged from the interviews. A number of boys experienced their detention period as “useless” and mentioned a lack interventions and activities to work on their (treatment) goals.

“*I haven't worked on anything. Since I put together an action plan, where… with things I needed to work on, that hasn't been looked at. That, that booklet hasn't even been opened since December*.”^27^ (Participant 148 at T3, 17 years old)

Furthermore, some of these boys experienced a mismatch between their needs and the goals they were working on, especially related to the primary needs of “excellence in work,” “excellence in play,” and “pleasure.” For example, some boys used to attend or wanted to attend higher education or art academy, types of education that the institutions typically do not provide. This mismatch could perhaps result in the feeling of “uselessness” as participant 148 only expressed his dismay at T3 after experiencing the mismatch he mentioned at T2 for a while.

“*They help you a… a little bit so, for example, I can do my art subjects here, such as 'perception drawing'. But other than that, it's like, they don't have computers, no strong computers that I can use for my art subjects. And uh, they only let me go [to my school] one day a week from January on… When actually one day is not going to be enough. So, on the one hand, they do make concessions, but on the other, they still kind of work against us*.”^28^ (Participant 148 at T2, 16 years old)

Others experienced a lack of leisure time activities:

"…*the only thing we do is, uh, play soccer for an hour and uh, watch TV. That's all we do in the group*.”^29^ (Participant 10 at T2, 17 years old)

Still, other boys had a more positive view of the activities arranged for them. Especially once the youth obtained more freedom (usually at T3 instead of T2), they viewed their detention as more positive and appear to be more motivated to work on their rehabilitation (see also the next section “Freedom and Autonomy: Excellence in Agency”).

“*On the weekends, we do fun activities and such. Last weekend, Saturday morning, I was outside with my mom for three hours. Three hours. Afternoon [we went] to the zoo. Sunday morning to the market, Sunday afternoon to the pool. It was a nice weekend*.”^30^ (Participant 3 at T2, 16 years old)

At T3 (compared to T2) we saw that more boys felt that being confined in the institution gave them the time and opportunities to reflect on their past and future.

“*Since I am here [in detention], I… This showed me, uh, showed me that uh, my degree and such is important*.”^31^ (Participant 2 at T3, 17 years old).

“*… my view on drugs has changed here. Now, yes, I just don’t want to use drugs anymore, because it messed up my life*.”^32^ (Participant 3 at T3, 16 years old)

They also viewed their time in detention as a useful way to work on their problems and goals.

“*Because yes, I do have a few problems, so… that I do need to work on. So, yeah. If I wasn't here, I wouldn't have gotten around to working on them. I'd still be… now just sitting at home and, yeah. I'm glad I can do something about it here*.”^33^ (Participant 133 at T2, 17 years old)

“*They really, yes the dreams that you have, like I say 'I want to be a firefighter, for example'…. Then they really go out of their way to make that happen, so that's something good*.”^34^ (Participant 133 at T2, 17 years old)

#### Freedom and autonomy: excellence in agency

Factors often mentioned as reasons for the boys’ diminished treatment motivation were related to restrictions on their freedom and autonomy (i.e., the primary good of “excellence in agency”). The boys perceived their lack of freedom as a restriction on their development and rehabilitation. This was especially true in the beginning of their detention (the first 2 months, before T2) when periods of leave and access to their mobile phones were not (yet) granted. They experienced being detained as hampering their possibilities to go (back) to school, work, and (re-)connect with their loved ones.

“*[I just miss…] the freedom. Too bad I'm sitting here. Everything is on hold too so to speak*.”^35^ (Participant 2 at T2, 17 years old)

“*I've been thinking a lot about what I want to do next, I'm so ready to get into action, but I just have to… If only I could start already… No, I can't, because I have to sit here for so long. Another month and a half.*”^36^ (Participant 3 at T2, 16 years old)

Some boys (usually in a later stage during detention at T3) also felt that although they had learned to change their behavior within the institution, their detention restricted them from practicing their new skills “outside.”

“*… if it [conflicts outside] would have happened and I would know of 'ah yes, that happened over the weekend and I got out of it like that', then I know of 'I can do it'. But, I haven't been able to try it yet, so yeah. I don't know…will I succeed. I don't know*.”^37^ (Participant 10 at T3, 17 years old)

Not only was their physical freedom restricted because of their detention; also in other ways the boys’ autonomy and privacy were restricted. For some this made it difficult to express themselves.

“*You do here, uh, what they ask. You never get to choose what you want to do yourself*.”(Participant 125 at T3, 16 years old)

“…*we also have no privacy, because if I call I have to call next to a staff member… because [last month] I had a very bad conversation…My stepmother had to cry. I was crying. Something bad had happened. Then the staff member is standing next to you… That's just not nice*.”^38^ (Participant 101 at T3, 18 years old)

“*[with more privacy…] you can be more, yes, like yourself. Because now… you have to… you behave according to what you think others want you to behave*.”^39^ (Participant 133 at T2, 17 years old)

Still, a few boys mentioned the beneficial deterring effect of strict rules and punishment. Being detained gave them insight into the fact that their behavior has consequences they wish to avoid.

“*Here, uh, you learn about reality. That if you do something, it won't just, uh, stay that way. You'll be punished anyway for what you do*.”^40^ (Participant 125 at T3, 16 years old)

About a third of the boys also experienced a lack of perspective on where to live after their release. They expressed feelings of insecurity about when they could leave and where they would reside.

“*That it took a long time to really have a perspective. Uh, because first they had said three months, then another three months…. Actually, they fooled me*”^41^ (Participant 87 at T3, 17 years old)

“*I have to go into assisted independent living. But uh, with the waiting lists I've heard that it can last until April*.”^42^ (Participant 148 at T2, 16 years old)

Conversely, having perspective, working towards more freedom and their own goals motivated the boys towards participation in rehabilitation.

“*You can go to school [outside the institution], uh, after three months. Being free as soon as possible… away from here, uh… Just, going away as soon as possible… that motivated me*.”^43^ (Participant 125 at T3, 16 years old)

#### Staff factors: relatedness through therapeutic alliance

On their path to rehabilitation, a majority of the boys mentioned the importance of the interactions they had with the professional staff. Building a strong, trusting, and positive therapeutic alliance (i.e., fulfilling the primary good of “relatedness”) appeared to benefit the boys’ motivation and rehabilitation efforts.

“*When they see something is wrong, they come to me and talk to me… I am happy that this is a good place, they do things like that.*”^44^ (Participant 133 at T2, 17 years old)

Contrarily, staff members who the boys experienced as unfriendly, arrogant, demanding, and punitive were deemed to decrease their treatment motivation and hamper their rehabilitation.

“*…some staff members here, I don't know what's wrong with them, but they seem to think to be more than us… They see us… like we're locked up here, like we're not people but animals. They assume the worst of us… They don’t know me as a person, yet they accuse me as a criminal*.”^45^ (Participant 166 at T2, 17 years old)

“*Ah they, abuse their power. You can't give your own opinion. They give you a comment… and if you give your opinion about it, they send you to your room*.”^46^ (Participant 27 at T3, 16 years old)

When asked what would help them in the interaction with staff, the boys mentioned they would want to be treated not as criminals, but as the person they are behind their behavior and the crimes they committed.

“*Gosh, just, not thinking that… that we're like… boys who have committed crimes or just boys who, I don't know, have problems at home, because… We're really just normal boys. We’re just people who made mistakes*.”^47^ (Participant 147 at T2, 17 years old)

Others mentioned that authentic curiosity, empathy, and transparent communication from the staff helped them to be more motivated to work with them on their rehabilitation goals.

“*Really just talking… Really understanding them. Just, giving more examples for them. What life really is like.*”^48^ (Participant 27 at T2, 15 years old)

“*And if they don't understand you, they should show us 'I want to understand you, I want to help you. I'm not here just to tell you what you can and can't do in here. I am here uh… to help you and I want you to stop committing crimes when you go outside.*'”^49^ (Participant 27 at T2, 15 years old)

#### Individual factors: inner peace and relatedness

Finally, factors related to inner peace and their social environment played an important role in the boys’ treatment motivation and rehabilitation as well. The most often mentioned individual factor that negatively affected their treatment motivation and rehabilitation was their inability to manage negative emotions, such as anger and stress (i.e., problems with the primary good of “inner peace”).

“*If I have to listen, but they say it too roughly or something, then… Yes. Then I deal with that badly, then I start yelling and get angry.*”^50^ (Participant 10 at T3, 17 years old)

“*Sometimes you can’t really get rid of your, your stress or your difficult…, your difficult things. Because you're here, you're trapped here…. and it keeps piling up. And after a while I just get to a certain point that I just snap. That I just start hitting a wall or something.*”^51^ (Participant 120 at T2, 17 years old)

Related to this, some youth experienced the impact of their (previous) substance abuse, such as cravings and withdrawal symptoms.

“*But I also think that that [getting angry] was also that I was going through a bit of a rehab…*”^52^ (Participant 60 at T2, 17 years old)

Whereas negative emotions and difficulties in coping could hamper rehabilitation, learning to cope with these emotions effectively (i.e., working on the primary good of “inner peace”) during treatment had a positive effect. Over time, building rapport and a relationship of trust with staff (i.e., attaining “relatedness”) could help the youth express their feelings in more adequate ways.

However, some youth experienced a lack of trust in the professional staff working at the institution, making it difficult for them to talk about their problems.

“…*I don't trust them [the staff]. I can't talk to them. I don't want to talk to them…with them I really have to watch what I say and stuff. That's not nice*.”^53^ (Participant 101 at T3, 18 years old)

In these cases, professionals from outside the institution could be helpful as the youth felt they could share experiences that would not impact their relationships with the staff and the reports to court.

“*… and they [mental health professionals from another institution] give me a safer feeling than the staff [here]. With them, I can relax. Just talk*.”^54^ (Participant 101 at T3, 18 years old)

A few boys mentioned the negative influence of antisocial friends they had back home and/or met in the institution.

“*You come in here, you meet a few people. You find a way to make money, when you're free you're going to do that, and if we get caught, we're just going to be in [detention centers] anyway and those institutions are actually not too bad.*”^55^ (Participant 3 at T2, 16 years old)

On the other hand, some boys managed to make new (prosocial) friends (i.e., attaining the primary good of “relatedness”) when they were allowed to go on leaves outside the institution, that had a beneficial effect.

“*Yes, certain people I don’t see anymore, but I wouldn’t want to. Uh, I have new friends as well. Uh, and yes, my other friends are just a lot happier now that I am back with them [when I am back home]*.”^56^ (Participant 5 at T3, 16 years old).

Finally, working on the relationship with family, especially parents (again, attaining the primary good of “relatedness”), was perceived as beneficial to their rehabilitation.

“*The bond with my mother has grown stronger… after everything that happened, not being able to see each other often and… facing my problems, it has improved.*”^57^ (Participant 133 at T2, 17 years old)

“*Just because uh, they [my family] mean a lot to me. And uh, I have disappointed those people, a lot, and now… now I want them… I want them to be proud of me so to say.*”^58^ (Participant 60 at T2, 17 years old).

### Antisocial and prosocial attitudes

All the interviews ended with a question about whether or not the boys would desist from crime and lead a prosocial life after their release. About half of the boys stated they did not want to part with their antisocial lifestyle. Almost all of these mentioned money as their main motive for this choice.

“*Interviewer: And why not quit altogether? Participant: That's difficult. Interviewer: And what … what makes it difficult? Participant: The money… And if I… there is a good deal, I will grab that anyway. I won’t let it slide. I won’t even have to think about it.*”^59^ (Participant 101 at T3, 18 years old)

The other half of the boys stated they would never commit any crimes again.

“*Because that's the most important thing to me: What I'm going to be later. To, yeah, I don't want to be a junkie, or anything, later, so yeah. Or a thief or I don't know, a criminal. So, I think my future is very important.*”^60^ (Participant 147 at T2, 17 years old)

They expressed that the reasons for choosing a prosocial life came either from a drive within themselves or what they had learned while being detained, or a combination of both.

“*I knew what I had done wrong, I knew what I should do better… I just knew from myself, yes 'I'm not going to do this again', I'm going to think before I do things in the future. And I didn't need this place for that*.”^61^ (Participant 148 at T3, 17 years old).

“*You also learn a lot in here and, yes. I also don't want to throw away everything I've learned from it, so… I think that [desisting from crime] is possible, yes.*”^62^ (Participant 133 at T2)

## Discussion

In this study we examined assumptions of the Good Lives Model (GLM) about primary goods satisfaction and well-being, and their links with treatment motivation and rehabilitation in young offenders. We analyzed 28 interviews conducted in a sample (*N* = 20) of arrested detained adolescents. The themes that emerged from the qualitative analysis consisted of self-perceived well-being needs, treatment goals (formulated by the institution and/or the youth themselves), and factors perceived to affect their treatment motivation and rehabilitation.

The results show that the adolescents’ well-being needs were linked to their treatment goals, set by themselves and/or the institutional staff. The needs and goals mentioned as important to the boys were related to the primary goods of “relatedness” with family and friends, “excellence in work,” “excellence in play,” “pleasure,” “excellence in agency,” “inner peace,” and “life” (the latter including their living and financial situation). The other four GLM primary goods (i.e., “knowledge,” “creativity,” “community,” and “spirituality”) were not mentioned by the boys in relation to their treatment needs and goals. This could infer that some goods may be more salient in detained adolescents, such as “relatedness” and “inner peace,” compared to “spirituality” or “creativity.” That is, previous studies address the idea that the GLM should be viewed as pluralistic and subjective in its emphasis on social, cultural, and individual differences in which primary goods are deemed more or less important, and the different ways individuals seek to achieve them (Ward and Marshall, 2007; Ward and Maruna, 2007; Serie, 2022).

### Matching needs and goals

When the boys experienced a match between their need for specific primary goods and the treatment goals that were adhered to in detention, they experience higher treatment motivation, and it benefited their rehabilitation process. A mismatch between them was perceived as detrimental. Specifically, the boys mentioned there was often a mismatch within their work and school needs and the goals they were working towards within the institution. They felt that being detained put their academic/work career on hold. On the other hand, boys who experienced their detention period as promoting their academic and career goals (i.e., “excellence in work”), expressed more treatment motivation. This finding is in line with previous studies that showed successful engagement in education (satisfying), lawful employment, and financial independence can effectively reduce recidivism (Wright and Cullen, 2004; Katsiyannis et al., 2008; Zagar et al., 2013; van den Berg et al., 2014; Hill et al., 2017; Niebuhr and Orrick, 2020). Thus, working towards prosocial career goals (outside the detention centers) could serve as a positive alternative to earning money through illegal means. Eventually, this way the boys can learn the skills to legally provide for themselves and find a place to live on their own (i.e., “life”) in the future. This seems to be particularly important for these youth, because for some of the boys in our study money seemed to be their main motive for their criminal offending.

Next, being granted more freedom and autonomy (i.e., “excellence in agency”) in and outside the detention centers also seemed beneficial in enhancing treatment motivation. Previous research showed that youth detention interventions tend to be highly structured and can be (experienced as) repressive (Schubert et al., 2012; van der Helm et al., 2014; Van der Helm et al., 2018; De Valk et al., 2019). Furthermore, these studies show that a repressive environment could restrict the youth’s (sense of) autonomy, reduce treatment motivation, and create resistance. To counteract this, the GLM encourages offenders to pursue their personal, prosocial goals and argues for intrinsically motivated change, rather than having it imposed from outside. By working together towards the offender’s personal goals and enhancing their self-efficacy, a sense of autonomy can be gained, even while being incarcerated (Mann et al., 2002; McMurran and Ward, 2004; Ward et al., 2006; van der Kaap-Deeder et al., 2017; Tyler et al., 2020). Our study showed that with more autonomy and freedom the boys felt more responsibility for their own choices, and they experienced a positive future perspective, which reportedly increased their treatment motivation.

Additionally, the findings of our study show the importance of enhancing the boys’ coping skills (i.e., “inner peace”). Previous studies conclude that youth who suffer from emotional trauma and internalizing symptoms (e.g., anxiety and depression) are more aware of their problems and experience more emotional discomfort, which can enhance their willingness to engage in treatment (DiGiuseppe et al., 1996; Leenarts et al., 2013; Van Damme et al., 2015; DiPierro-Sutton et al., 2021). In comparison, externalizing disorders (e.g., conduct disorder) and symptoms of severe mental illness (e.g., psychotic disorder) are known to hinder treatment engagement, due to a lack of problem insight, social adjustment problems, interpersonal distrust and authority issues (DiGiuseppe et al., 1996; van Binsbergen et al., 2001; Roedelof et al., 2013; Brooks and Khan, 2015).

In line with this, a majority of the boys in our sample struggled with negative emotions, such as stress, grief, and anger. In these cases, professional psychological help from external parties outside the institution could be helpful. This was especially the case for youth who have difficulty trusting institutional staff members who are linked to the juvenile justice system. It is well-known that establishing a therapeutic alliance in offender rehabilitation can be challenging due to the dual-role issues faced by professionals working in (juvenile) justice systems. Professionals who work in the field of (juvenile) justice often find themselves in the dilemma between helping “patients” and protecting society from “offenders.” That is, on the one hand they have the task of helping the youth in their rehabilitation process. On the other hand, they also advise the court about their recidivism risk and need for treatment (Ward, 2013; Barnao et al., 2016). Furthermore, (adolescent) offenders might react distrustful, defensive, and oppositional to authorities and the mandated interventions imposed upon them, especially when they suffer from psychopathology, trauma, and stigma (Wittouck and Vander Beken, 2019).

Furthermore, within the detention regime, it is important to promote a strong, trusting, and positive therapeutic alliance with staff members within the institutional environment (i.e., “relatedness”). A positive therapeutic alliance has been linked to higher levels of treatment motivation, positive behavior change, and less re-offending (risk), in both adult and adolescent offenders (Florsheim et al., 2000; Marshall et al., 2003; Holmqvist et al., 2007; Polaschek and Ross, 2010; Ward and Laws, 2010; Harder et al., 2012; Hughes, 2012; Hart and Collins, 2014; Roest et al., 2016; Hachtel et al., 2019; Van Hecke et al., 2021). In our study, staff members who were perceived as empathic, curious, unprejudiced, supportive, and transparent in their communication were experienced as contributing to the boys’ treatment motivation and rehabilitation. These professionals could (also) support the youth in teaching them more effective coping skills by offering a listening ear and modeling more helpful coping strategies.

Finally, working on prosocial relationships with friends and family (as alternatives to their relationships with antisocial peers; i.e., “relatedness”) was viewed as an essential element in treatment motivation and rehabilitation. Contact with antisocial peers was perceived as a strong influence on the boys’ behavior. Boys who could rely on alternative, prosocial others outside the institution and back home felt it was easier to resist antisocial influences. Thus, a focus on attaining the primary good of “relatedness” through positive relationships with family and friends is deemed an important treatment goal for effective rehabilitation. An extreme example to foster such change in social contacts is moving to a different neighborhood, which has been found to be an effective turning point in reducing recidivism (Hoeve and van der Laan, 2016). Other (less extreme) examples consist of joining associations and sports clubs, and/or changing schools. Focusing on prosocial contacts automatically diminishes the engagement with antisocial peers (Warr, 1993; Dishion and Tipsord, 2011). Furthermore, it may also be beneficial to work on improvement of social skills and assertiveness, especially when confronted with (antisocial) peer pressure they wish to avoid (Lipsey et al., 2010; Tracey and Hanham, 2015).

### Primary goods and/or risk factors?

What is striking in our results, is the considerable similarity between the most important criminogenic needs of the RNR model (the “central eight,” mainly criminal peers, family/family problems, school/work problems, lack of constructive leisure activities, and substance abuse) and the primary goods of the GLM (“relatedness,” “excellence in work and play,” and “inner peace”; Looman and Abracen, 2013; Serie, 2022).

As a result, important life domains (relationships with family and friends, school/work, leisure, and cognition/emotions) can both be addressed in accordance with the RNR model by reducing related criminogenic risks, and in accordance with the GLM by focusing on the prosocial attainment of the primary goods (Ward and Stewart, 2003a,b,c; Serie, 2022). Thus, combining the RNR model with the GLM could enhance each other, both from their own vantage point (Ward, 2002a,b; Ward and Maruna, 2007).

In addition, although the RNR model underlines the importance of treatment motivation and responsivity (Bourgon and Bonta, 2014), it lacks a comprehensive framework or guidelines on how to implement it in practice. Apart from briefly mentioning motivational interviewing as a possible responsivity technique to increase treatment motivation, the RNR model does not elaborate on how to effectively engage offenders in treatment, while the GLM does (Andrews and Bonta, 2010; McMurran and Ward, 2010; Bonta and Andrews, 2017). That is, focusing not only on risk reduction, but also adhering to the offenders’ self-determined personal goals for their well-being (i.e., primary goods) can increase their treatment motivation (Ward and Fortune, 2013).

### Enhancing motivation to desist

Taken together, our results show that the primary goods the youth deemed most important for treatment motivation and rehabilitation are reflected in the primary goods that are related to the psychological needs for motivation and well-being from self-determination theory (SDT): “excellence in work and play” (in SDT terms: “competence”), “excellence in agency” (in SDT terms: “autonomy”), and “relatedness” (Deci and Ryan, 2000; Ryan and Deci, 2017a).

SDT approaches motivation as a continuum, ranging from a complete lack of motivation or “a-motivation”; through “extrinsic motivation” that is externally regulated by outside pressure or control; to “intrinsic motivation,” which originates within the self, is fully autonomous and self-determined (Deci and Ryan, 2000; Ryan and Deci, 2000, 2017a). These different types of motivation resonate with Prochaska and DiClemente’s (1986) stages of change model used in Motivational Interviewing. The stages of change model argues that within the process of behavior change people generally progress from a “precontemplation stage” (“a-motivation”), through a “contemplation” and “action stage” (“extrinsic motivation”), to long term “maintenance” of the changes (“intrinsic motivation”; Prochaska et al., 1992; McMurran and Ward, 2004). Accordingly, behavior change that is extrinsically motivated will most likely only last as long as the extrinsic controls are in place. On the other hand, intrinsic motivation is related to maintenance of sustainable change (Deci and Ryan, 2000, 2008; Ryan and Deci, 2017a). In this reasoning, an offender who is only extrinsically motivated to desist from crime because of the risk of incarceration and parole requirements, will have a higher chance to recidivate than an offender who is intrinsically motivated to leave their criminal lifestyle behind.

Based on this conceptualization of motivation by SDT, the GLM focus on enhancing *intrinsic motivation* for behavior change. To accomplish this goal, the GLM aims to promote prosocial treatment goals that are personally relevant to the offender and their well-being. Likewise, in line with the SDT, the GLM emphasizes the importance of pursuing and achieving goals that are intrinsically meaningful (i.e., related to the basic psychological needs and GLM’s primary goods) instead of extrinsically controlled or imposed (Ward et al., 2006; Tyler et al., 2020).

In accordance with our results, the satisfaction of the SDT’s three psychological needs (“competence,” “autonomy,” and “relatedness”) has previously been linked to higher levels of intrinsic motivation, treatment engagement, and positive behavior change (Milyavskaya and Koestner, 2011; Ng et al., 2012; Jochems et al., 2016; Ryan and Deci, 2017a; Hope et al., 2019; Rodríguez-Meirinhos et al., 2020). Moreover, in adult offenders, psychological need satisfaction has been related to higher levels of quality of life, intrinsic motivation, and persistence to desist from crime (van der Kaap-Deeder et al., 2017; Petrich, 2020). Consequently, some of the primary goods of the GLM (i.e., “excellence in agency,” “excellence in work and play,” and “relatedness”) may be more important than others for improving treatment motivation.

### Limitations and recommendations

In the current study we aimed to examine the GLM’s assumptions about primary goods satisfaction and well-being and its effects upon treatment motivation and young offender rehabilitation based on semi-structured interviews with detained adolescents. However, when relying on self-report and qualitative methods, particularly in detainees, some problems may arise that threaten the reliability and validity of the information.

First, the interviews were coded by a single researcher due to practical reasons, which prevents assessing the (interrater) reliability of the analysis. In addition, although we asked the boys who participated in the larger study at random if they wanted to participate in the interview, self-selection may have occurred as only about half of the boys who were selected agreed to participate. That is, the youth who were willing to participate likely were also the ones who could more easily express themselves. Still, additional attrition analyses^63^ comparing the current subsample to those who did not participate in the interview revealed no substantive differences (in demographics, well-being measures and delinquency rates).

Second, the participants could be answering in a socially desirable manner, unwilling to talk (truthfully) about their (antisocial) views and attitudes. This can be especially the case for offenders who are awaiting their trial at the beginning of their detention period (Bernasco, 2013). Therefore, to elicit truthful statements as much as possible, the interviewer emphasized anonymity of the interviews and survey data throughout the studies. Moreover, investing in rapport-building from the start of the study was a key component in obtaining sincere disclosures. As a result, most participants seemed genuinely pleased to talk about their experiences. Nevertheless, their answers only reflect the boys’ perceptions, opinions, and attitudes, which may or may not reflect their actual (future) behavior. Additionally, as the participants had already been detained for a while, over time their answers may have been influenced by their memories and/or by conversations they previously had with the judge, other (juvenile justice) professionals, parents, other family members, and peers.

Finally, we only followed the youth for a relatively short period during detention (up to 6 months after the boys’ arrest). Future studies are recommended to follow these youth for longer periods of time during and after detention, collecting both quantitative (e.g., including recidivism rates) as well as qualitative data. In this way the data could reveal which factors during detention affect long-term outcomes after detention.

## Conclusion

The current qualitative analysis revealed relevant insights into the views on treatment motivation and effective rehabilitation from the individual’s perspective. The results show that a match between the boys’ well-being needs, and the treatment goals (in collaboration) within the institution could improve self-perceived treatment motivation and rehabilitation efforts. Other factors mentioned by the boys to positively affect their treatment motivation include: an increase in freedom and autonomy, having a future (prosocial) perspective, investing in a therapeutic alliance, and working on individual factors (i.e., developing coping skills, school or work skills, and relationships with prosocial friends and family). These factors closely align with working on the GLM primary goods that are related psychological needs of the Self-Determination Theory: “excellence in work and play,” “excellence in agency,” and “relatedness,” which can be helpful in enhancing well-being and treatment motivation in offender rehabilitation. More research is recommended to examine more long-term effects of working on GLM primary goods in detention, with both qualitative and quantitative measures.

## Data availability statement

The raw data supporting the conclusions of this article will be made available by the authors, without undue reservation.

## Ethics statement

The studies involving human participants were reviewed and approved by Social and Societal Ethics Committee of the Catholic University of Leuven (G-2017 10,945). Written informed consent from the participants’ legal guardian/next of kin was not required to participate in this study in accordance with the national legislation and the institutional requirements.

## Author contributions

CS is the main author of this publication. CR, SP, and JP provided supervision and feedback in this PhD project. All authors contributed to the article and approved the submitted version.

## Funding

This research was supported by FWO (research project grant G073018N).

## Conflict of interest

The authors declare that the research was conducted in the absence of any commercial or financial relationships that could be construed as a potential conflict of interest.

## Publisher’s note

All claims expressed in this article are solely those of the authors and do not necessarily represent those of their affiliated organizations, or those of the publisher, the editors and the reviewers. Any product that may be evaluated in this article, or claim that may be made by its manufacturer, is not guaranteed or endorsed by the publisher.

## Footnotes

^1^The current study received ethical approval as part of a larger study (Serie, 2022) from the director of the Flemish Youth Detention Centers, the Dutch Custodial Institutions Agency (Dienst Justitiële Inrichtingen), and the Social and Societal Ethics Committee of the Catholic University of Leuven (G-2017 10,945).

^2^“Met mijn vader zo praten. Gewoon het thuis zijn….”

^3^“De weekendbezoeken. Of als mijn… mijn vriendin hier komt, op bezoek. […]. Dan vergeet… dan vergeet ik zo al de rest.”

^4^“Hm… Gewoon de band met mijn familie… Ik hoop dat die verbetert. En als ik terug thuis ben dat dat goed gaat worden. De band met mijn vader wil ik ook zeker verbeteren. Euh… Die was jarig, vorige zondag… Ik wou hem bellen, maar ik heb blijkbaar een contactverbod met hem ineens.

^5^“[Ik ben blij…] nu, als ik thuis ben of, euh, als ik… mijn vrienden weer zie. Euh… Ja. Nu is dat… Nu vind ik dat wel raar om te zeggen want dat was nooit met zo ‘[ik ben blij…] als ik mijn moeder blij zie’ bijvoorbeeld.”

^6^“Ik wil gewoon naar school… Omdat ik weet dat ik het kan en ik weet da tik me moet bewijzen bij de rechtbank.”

^7^“Ik denk het enigste dat goed gaat is dat ik gewoon nog steeds op school ben ingeschreven. Dat ik daar niet van geschorst word.”

^8^“Ik ben wel aan het denken om andere voetbalclub of ja, gewoon sowieso, te voetballen als ik hier weg ben.”

^9^Participant: “… dat moet gewoon een open instelling zijn.” Interviewer: “Waarom zou dat helpen denk je? Als dat open is? Participant: “Want geen enkel kind of jongere wilt opgesloten zijn.”

^10^“Gewoon minder… minder regels. Meer vrijheid.”

^11^“Ik wil gewoon dat euh… Ik ben weer vrij, ik wil gewoon die dag… wil ik zeg maar genieten dat ik gewoon vrij ben. En wil ik dat ik niet euh, hoe zeg je dat, dat ik niet wil dat mensen tegen mij zeggen ‘om half tien moet je in je cel’.”

^12^“…maar belangrijker is uw eigen keuzes maken. Uw eigen leven bepalen.”

^13^“Maar er is sowieso altijd nog iemand die nog kan beslissen over u, dus… Ge moet toch deels wel luisteren en wel ook nog zelf beslissingen kunnen nemen.”

^14^“…als ge smoort… dan kan niks u schelen zeg maar. Alee niks… minder.”

^15^Interviewer: “Je zei ook dat je een goed leven voor hierbuiten wilt, wat bedoel je daar precies mee?”

^16^“Ik wil mijn diploma halen. En euh, nu dat ik alleen ga wonen, wil ik me ook euh, wil ik ook werken enzo.”

^17^“Als ge rijk zijt dan kunt ge gewoon meer dingen doen. Meer dingen betalen, gewoon… Dan leeft ge toch iets luxer dan… een normale persoon.”

^18^“Ja, ja dat moet nu wel. Alee ja, daar aan werken… Euh… Ik moet nu naar huis gaan op die dag-bezoeken enzo.”

^19^En euh, dan in het weekend met die dag-bezoeken bijvoorbeeld, dan ga ik niet, niet met vrienden rondhangen ofzo. Ik euh, probeer gewoon zo veel mogelijk bij mijn ouders te zijn. …allee, ik ben niet altijd bij mijn ouders, ik ben ook vaak alleen met mijn vriendin, maar dan probeer ik wel ook sowieso met mijn, euh, met mijn moeder iets te gaan doen ofzo. Gelijk vorig weekend ben ik met mijn moeder en mijn vriendin dan iets gaan eten.

^20^“Ik zou die graag aan de kant willen schuiven en opnieuw willen beginnen met nieuwe vrienden, maar… Dat is niet zo gemakkelijk, want ik ken die ook al… veel vrienden, vier, vijf jaar….”

^21^“Euh, belankrijk [voor mij]… Ja nu, dat ik mijn school afmaak. Mijn school afmaken en werk vinden.*”*

^22^“[Ik werk aan]… aan euh, hoe ik op slechte situaties het beste kan reageren. In plaats van in de aanval over te gaan, hoe ik een betere oplossing kan zoeken zonder in de aanval te gaan.”

^23^“Dat ik na… na leer denken over de dingen dat ik vroeger beter anders had kunnen doen… en dat ik ze in de toekomst ook anders moet doen. En, dat je daar hier nu ook bij stil moet staan, omdat je ja, je zit daar [in detentie] hiervoor.”

^24^“Als ik mij slecht voel laat ik dat zien in mijn gedrag, maar ik praat niet echt.”

^25^“…gewoon door met de opvoeders en sociale dienst en psycholoog enzo, over te praten van kijk dit heb ik… dit gaat er mis in mijn leven….”

^26^“Ze [de instelling] hebben dat [begeleid zelfstandig wonen] voorgesteld, maar ik vind dat zelf ook belangrijk. Als ik achttien ben of ouder dat ik zelfstandig kan zijn.”

^27^Ik heb aan niks gewerkt. Sinds dat ik een actieplan heb opgesteld, waar… met dingen waar ik aan moest werken, is daar niet meer naar gekeken. Dat, dat boekje is niet eens open geweest sinds december.

^28^“Die helpen jou een… een beetje zo, bijvoorbeeld hé dat ik mijn kunstvakken hier kan doen, zo waarnemingstekenen. Maar voor de rest, is dat zo van, ze hebben geen computers, geen sterke computers die ik kan gebruiken voor mijn kunstvakken. En euh, ze laten mij maar één dag gaan vanaf januari… Terwijl eigenlijk één dag niet voldoende gaat zijn. Dus, voor de ene kant, ze doen wel toegevingen, maar voor de andere kant, werken ze toch nog steeds een beetje tegen.”

^29^“…het enigste wat we doen is, euh, een uur voetballen en euh, tv kijken. Dat is het enigste wat we doen in de leefgroep.”

^30^“In de weekenden doen wij leuke activiteiten enzo. Vorig weekend, zaterdagvoormiddag, was ik drie uur met mijn mama buiten. Drie uur. Namiddag [gingen we] naar de zoo. Zondagochtend naar de markt, zondagnamiddag naar het zwembad. Het was wel een leuk weekend.”

^31^“Sinds ik hier zit heb ik zo… Dit heeft mij zo meer, euh, doen laten inzien dat, euh, diploma enzo belangrijk is.”

^32^“… mijn visie op drugs hier wel veranderd is. Nu, ja, ik wil gewoon geen drugs meer gebruiken, want dat heeft mijn leven wel echt verkloot.”

^33^“Want ja, ik heb wel een paar problemen, dus… waar ik wel aan moet werken. Dus, ja. Als ik hier niet was, was het er ook niet van gekomen dat ik er aan werkte. Had ik nog… nu gewoon thuis gezeten en, ja. Ik ben wel blij dat ik er iets aan kan doen hier.”

^34^“Ze gaan echt, ja de dromen dat ge hebt, gelijk, ik zeg ‘ik wil brandweer worden bijvoorbeeld’… Dan gaan ze echt alles er aan doen om dat waar te maken, dus dat is wel iets goed.

^35^“[Ik mis…] gewoon de vrijheid. Spijtig dat ik hier zit. Alles staat ook op een pauze zeg maar.”

^36^“Ik heb veel nagedacht over wat ik hierna wil doen, ik ben zo klaar om in actie te komen, maar ik moet nu gewoon… Kon ik maar daar nu al aan beginnen… Nee, dat gaat niet, want ik moet hier nog zo lang zitten. Nog anderhalve maand.”

^37^“… als het [conflicten buiten] zou gebeurd zijn en ik zou weten van ‘ah ja, dat is gebeurd in het weekend en ik ben er zo vanaf gekomen’, dan weet ik van ‘ik kan het’. Maar, ik heb het nog niet kunnen proberen, dus ja. Ik weet niet van…lukt het wel. Dat weet ik niet.”

^38^“…we hebben ook geen privacy, want als ik bel dan moet ik naast de opvoeder bellen… want ik had een heel erg gesprek… Mijn stiefmoeder moest huilen. Ik moest huilen. Er was iets ergs gebeurd. Dan staat die opvoeder naast jou… Dat is gewoon niet leuk.”

^39^“[met meer privacy…] dan kunt ge meer, ja, uzelf zijn. Want nu… ge moet… ge gedraagt u naar wat ge denkt dat anderen willen dat ge u gedraagt.”

^40^“Hier, euh, leert ge realiteit. Dat als ge iets doet, dat dat niet zo maar, euh, zo, zo gaat blijven. Ge wordt sowieso wel gestraft voor wat ge doet.”

^41^“Dat het lang geduurd heeft voordat er echt een perspectief was. Euh, omdat ze eerst hadden gezegd drie maanden, dan nog is drie maand…. Eigenlijk hebben ze mij voor de zot gehouden.”

^42^“Ik moet begeleid zelfstandig gaan wonen. Maar euh, met wachtlijsten heb ik gehoord dat tot april duren.”

^43^“Ge moogt naar school gaan, euh, na drie maanden. zo snel mogelijk vrij… hier weg van, euh… Gewoon, zo snel mogelijk weg zijn hé… dat heeft mij gemotiveerd.”

^44^“Als ze zien dat er iets is komen ze naar mij en ze praten met mij… dat ik wel blij ben en dat dat een goeie plek is hier. Dat ze echt zo iets doen.”

^45^“…sommige opvoeders hier, ik weet niet wat er met hun is, maar ze voelen ons precies meer dan ons… Ze zien we zitten… we zitten hier vast, alsof we geen mensen zijn maar dieren. Ze gaan van ons slechtste uit… Ze kennen mij niet als persoon, maar toch verwijt ge mij als een crimineel.”

^46^“Ah die, misbruiken hun macht. Ge kunt uw eigen mening niet geven. Die geven uw een opmerking… en als ge uw mening daarover geeft, dan sturen ze uw naar de kamer.”

^47^“Goh, gewoon, niet denken dat… dat we zeg maar… jongens zijn die feiten hebben gepleegd of gewoon jongens die, weet ik veel, thuis problemen hebben, want… We zijn echt gewoon normale jongens. We zijn gewoon mensen die fouten hebben gemaakt.”

^48^“Echt gewoon praten… Ze echt begrijpen. Gewoon, meer voorbeelden geven voor hen. Hoe het leven echt in… in elkaar zit.”

^49^“En als ze u niet begrijpen moeten die laten zien van ‘ik wil u begrijpen, ik wil u helpen. Ik ben hier niet alleen om te zeggen wat kan en wat niet kan hierbinnen. Ik ben hier euh… om u te helpen en ik wil dat ge geen feit meer pleegt al ge buiten komt.’”

^50^“Als ik moet luisteren, maar die zeggen dat te ruw ofzo, dan… Ja. Dan ga ik daar slecht mee om, dan ga ik roepen en word ik boos.”

^51^“Je kan soms niet echt je, je stress of je moeilijke, je moeilijke dingen kwijt. Want je zit hier, zit hier gevangen… en het blijft opstapelen. En na een tijd kom ik gewoon tot een bepaald punt, dat ik gewoon uitbarst. Dat ik gewoon tegen een muur begin te slaan ofzo.”

^52^*“*Maar ik denk ook dat dat ook was dat ik een beetje aan het afkicken was….”

^53^“…ik vertrouw hun niet. Ik kan niet met hun praten. Ik wil ook niet met hun praten… bij hun hun moet ik echt opletten wat ik zeg enzo. Dat is niet leuk.”

^54^“En die geven mij ook een veiliger gevoel dan die opvoeders. Bij hun, ik kan effe rustig zijn. Gewoon praten.”

^55^“Je komt hier binnen, ge ontmoet een paar mensen. Jullie vinden een manier om geld te maken, als je vrij bent gaan jullie dat doen, en als we gepakt worden dan komen wij toch gewoon in [detentie instellingen] en die instellingen vallen eigenlijk nog wel goed mee.”

^56^“Ja, ik zie bepaalde mensen niet meer, maar dat wil ik ook niet. Euh, ik heb nieuwe vrienden ook. Euh, en ja, mijn andere vrienden zijn gewoon veel blijer nu dat ik terug ben bij hen.”

^57^“Mijn band met mijn moeder is nog sterker geworden… na alles wat er gebeurd is, elkaar niet vaak zien en… mijn problemen eigenlijk onder ogen zien, is dat wel beter geworden.”

^58^“Gewoon omdat euh, zij betekenen heel veel voor mij. En euh, ik heb die mensen vaak, veel teleurgesteld eigenlijk en nu… nu wil ik die… dat die trots zijn op mij zeg maar.”

^59^“Interviewer: En waarom zou je er niet helemaal mee stoppen? Participant: Dat is moeilijk. Interviewer: En wat… wat maakt het moeilijk? Participant: Het geld…En als er een goede deal is, ik pak die sowieso. Ik laat die niet staan. Ik ga er zelfs niet nadenken over.”

^60^“Omdat dat voor mij de belangrijkste is. Wat ik later ga worden. Om, ja, ik wil later geen junkie ofzo worden, dus ja. Of een dief of een weet ik veel, of crimineel. Dus, ik vind mijn toekomst heel belangrijk.”

^61^“Ik wist wat ik verkeerd gedaan had, ik wist hoe ik het beter zou moeten doen…Ik wist gewoon van mezelf, ja ‘dit ga ik niet meer doen’, ik ga nadenken voordat ik dingen ga doen in het vervolg. En daar had ik deze plek niet nodig.”

^62^“Ge leert hier ook wel veel en, ja. Ik wil ook niet alles wegsmijten wat dat ik ervan geleerd heb, dus… ik denk wel dat dat kan ja.”

^63^Not included in this article but on request available from the first author.

## Appendix A

**Semi-structured interview guideline:**

Before we begin I would like to emphasize that you are not obliged to answer everything, but of course you help me the most by telling as much as you can honestly. Everything is anonymous, which means that no one except myself will know what you say here. The only exception to this is that if there was an imminent serious danger to someone I would have to report it.

If it is OK with you I will record the conversation, but once I have processed everything anonymously I will delete the recording, is that OK?

Last time we talked in an interview about things you think are important, what makes you happy/joyful and what caused you problems.Now I want to ask you some questions about what it is like in here, whether your views have remained the same or maybe things have changed.

**– How do you like it here?**

→*Why?*
**– What are you working on, here in the institution?**
→*How?*
→*How do you feel/think about that?*
→*What would you like to work on yourself?*
**– Is there something you think is good about being here?**
→*Why?*
**– Is there something you think is not good about being here?**
→*Why?*
→*What would need to be different according to you? How?*
**– Are you motivated to be here?**
→*Why? How come?*
→*What would help you to be (more) motivated?*
**– What is important for you in your life at this point?**
→*Why?*
→*How do you work on that?*
→*Did you work on that during your stay here? How?*
**– What makes you happy?**
→*What does that mean to you?*
→*How does it make you happy?*
→*How is that here in the institution?*
**– Are there certain things you wish to achieve? Do you have dreams or goals?**
→*How do you work on those? What do you do to aim for them?*
→*Did you work on that during your stay here? How?*
**– Do you think the treatment here is going to help you to stay out of trouble?**
→*Why?*
→*What would need to happen to help you stay out of trouble? How would that help?*
